# Infected tracheal diverticulum: a rare association with alpha-1 antitrypsin deficiency* **

**DOI:** 10.1590/S1806-37132014000600011

**Published:** 2014

**Authors:** Cecília Beatriz Alves Amaral, Sónia Silva, Salvato Feijó

**Affiliations:** Hospital de Santa Maria, Northern Lisbon Hospital Center, Department of Internal Medicine, Lisbon, Portugal. Department of Internal Medicine, Northern Lisbon Hospital Center, Hospital de Santa Maria, Lisbon, Portugal; Santarém Hospital, Santarém, Portugal. Santarém Hospital, Santarém, Portugal; Hospital de Santa Maria, Northern Lisbon Hospital Center, Lisboa, Portugal. Northern Lisbon Hospital Center, Hospital de Santa Maria, Lisboa, Portugal

**Keywords:** Tracheal diseases, Pulmonary emphysema, Diverticulum, alpha 1-antitrypsin deficiency

## Abstract

Tracheal diverticulum, defined as a benign outpouching of the tracheal wall, is rarely diagnosed in clinical practice. It can be congenital or acquired in origin, and most cases are asymptomatic, typically being diagnosed postmortem. We report a case of a 69-year-old woman who was hospitalized after presenting with fever, fatigue, pleuritic chest pain, and a right neck mass complicated by dysphagia. Her medical history was significant: pulmonary emphysema (alpha-1 antitrypsin deficiency); bronchiectasis; and thyroidectomy. On physical examination, she presented diminished breath sounds and muffled heart sounds, with a systolic murmur. Laboratory tests revealed elevated inflammatory markers, a CT scan showed an air-filled, multilocular mass in the right tracheal wall, and magnetic resonance imaging confirmed the CT findings. Fiberoptic bronchoscopy failed to reveal any abnormalities. Nevertheless, the patient was diagnosed with tracheal diverticulum. The treatment approach was conservative, consisting mainly of antibiotics. After showing clinical improvement, the patient was discharged.

## Abstract (pt)

Divertículos da traqueia são evaginações benignas da parede traqueal e raramente diagnosticados na prática clínica. Podem ser congênitos ou adquiridos, e na maioria dos casos são assintomáticos, sendo tipicamente diagnosticados em estudos post-mortem. Relatamos o caso de uma mulher de 69 anos que foi hospitalizada após apresentar febre, fadiga, dor torácica pleurítica e uma massa cervical à direita complicada por disfagia. Tinha antecedentes pessoais de enfisema pulmonar (deficiência de alfa-1 antitripsina), bronquiectasias e tireoidectomia. Ao exame físico apresentava murmúrio vesicular diminuído, hipofonese cardíaca e um sopro sistólico. Laboratorialmente apresentava marcadores inflamatórios elevados, e uma TC mostrou uma massa aérea, multiloculada na parede direita da traqueia, achados confirmados por ressonância magnética nuclear. Realizou ainda uma fibrobroncoscopia que se revelou normal. Assumiu-se o diagnóstico de divertículo da traqueia. O tratamento proposto foi conservador, consistindo principalmente de antibioticoterapia. Após melhora clínica, a paciente recebeu alta.

## Introduction

A tracheal diverticulum is a benign outpouching of the tracheal wall that is rarely diagnosed in routine clinical practice, and there are few reports of cases in the literature. Tracheal diverticula can be single or multiple^(^ 1 ^-^ 9 ^)^ and can present either as ovoid pedunculated cysts or sessile formations, their longest axis measuring up to 3 cm, communicating with the trachea by channels.^(^ 9 ^)^ Concerning etiology, tracheal diverticula can represent congenital anomalies of the tracheobronchial tree or acquired outpouchings from a weakened tracheal wall,^(^ 1 ^,^ 2 ^,^ 4 ^,^ 5 ^,^ 8 ^-^ 10 ^)^ the two conditions differing mainly in relation to the site of implantation and the histological features of the wall.^(^ 3 ^,^ 6 ^,^ 11 ^)^ Congenital tracheal diverticula are usually smaller and feature a narrower communication with the trachea than do those that are acquired. ^(^ 1 ^-^ 3 ^,^ 5 ^,^ 8 ^,^ 9 ^,^ 11 ^-^ 13 ^)^ The former are frequently right-sided, occurring 4-5 cm below the vocal cords or a few centimeters above the carina,^(^ 3 ^,^ 4 ^,^ 13 ^-^ 15 ^)^ and are histologically similar to the tracheal wall, comprising respiratory epithelia, smooth muscle, and cartilage, whereas acquired tracheal diverticula are composed exclusively of respiratory epithelia.^(^ 3 ^,^ 11 ^)^ The lack of cartilage in the acquired form is thought to be due to its origin, because it is caused by increased intra-luminal pressure or by the weakening of the structures after surgical procedures,^(^ 10 ^,^ 13 ^)^ resulting in evagination of the mucous membrane through vulnerable points in the trachea.^(^ 2 ^,^ 3 ^,^ 9 ^,^ 11 ^,^ 12 ^)^ Therefore, acquired tracheal diverticula can appear at any level, although they typically occur along the right posterolateral wall^(^ 6 ^,^ 16 ^,^ 17 ^)^ near the thoracic inlet.^(^ 1 ^,^ 2 ^,^ 8 ^)^

Tracheal diverticula are usually asymptomatic, and their frequency can therefore be underestimated.^(^ 5 ^,^ 7 ^,^ 18 ^)^ In most cases, the diagnosis is made on the basis of features observed on X-rays, on CT scans,^(^ 7 ^,^ 16 ^,^ 17 ^)^ during bronchoscopy,^(^ 13 ^,^ 16 ^)^ or in autopsies.^(^ 1 ^-^ 3 ^,^ 5 ^-^ 8 ^,^ 11 ^,^ 12 ^,^ 19 ^)^ Consequently, the reported incidence varies depending on the diagnostic tool used. For example, in a postmortem study, the incidence was estimated to be approximately 1%,^(^ 1 ^,^ 4 ^,^ 6 ^-^ 9 ^,^ 11 ^,^ 13 ^,^ 14 ^)^ compared with 0.3% in children diagnosed through bronchoscopy.^(^ 7 ^,^ 8 ^,^ 14 ^)^ However, in a study employing cervical CT, Buterbaugh & Erly estimated that paratracheal air cyst (i.e., tracheal diverticulum) occurs in approximately 3.7% of the population.^(^ 20 ^)^

Although infrequent and nonspecific, symptoms can develop in individuals with tracheal diverticulum.^(^ 3 ^)^ Because the diverticula usually act as reservoirs for respiratory secretions, they are sometimes associated with chronic cough and can become infected.^(^ 2 ^-^ 5 ^,^ 8 ^,^ 9 ^,^ 11 ^,^ 14 ^)^ Although less common, dyspnea, dysphagia, dysphonia, recurrent nerve paralysis, cervical neck swelling, hematemesis, and hemoptysis have been described in patients with tracheal diverticula.^(^ 2 ^,^ 8 ^,^ 12 ^)^ Tracheal diverticulum can also be associated with abnormal pulmonary function and obstructive lung disease,^(^ 7 ^-^ 9 ^)^ mainly emphysema,^(^ 4 ^,^ 20 ^)^ although no relationship between tracheal diverticulum and alpha-1-antitrypsin deficiency has been described to date.

In establishing a diagnosis of tracheal diverticulum, CT plays a fundamental role, providing information concerning the location, origin, and size of the lesion, thus helping distinguish between the congenital and acquired forms. Fiberoptic bronchoscopy, although useful, can yield false-negative results, because it often misses the communication point with the trachea. ^(^ 3 ^,^ 4 ^,^ 6 ^,^ 10 ^,^ 11 ^,^ 14 ^,^ 17 ^)^

Various approaches to the treatment of tracheal diverticulum have been described, including surgical resection, endoscopic cauterization, and conservative measures. Most tracheal diverticula are managed conservatively. Surgery is generally reserved for larger diverticula or truly symptomatic presentations with recurrent infections.^(^ 1 ^,^ 3 ^,^ 13 ^)^ Conservative management with antibiotics, mucolytics, and physiotherapy can be appropriate for asymptomatic, elderly, or debilitated patients.^(^ 2 ^,^ 3 ^,^ 11 ^,^ 14 ^)^ Here, we describe the case of a patient presenting with symptoms attributed to a tracheal diverticulum. 

## Case report

A 69-year-old woman presented to the emergency room with a one-week history of fever, fatigue, and pleuritic chest pain. After clinical examination, she was discharged on a one-week course of amoxicillin-clavulanate and azithromycin. Despite the antibiotic therapy, she showed no clinical improvement over the following days, and, rather than getting better, she noticed a painless right neck mass complicated by dysphagia. In addition, detailed history-taking revealed a two-month history of hoarseness with no other associated complaint. Consequently, she was admitted to the hospital.

Her medical history was significant: pulmonary emphysema secondary to alpha-1 antitrypsin deficiency (PiZZ phenotype); bronchiectasis; recurrent pneumonia (at least two episodes per year); a thyroid neoplasm treated surgically (by thyroidectomy) in 2001; hypertension; and a herpes zoster infection. She reported no alcohol abuse or history of smoking. Her usual medications included levothyroxine, valsartan, hydrochlorothiazide, and bisoprolol. 

On physical examination, she was afebrile with apparently normal skin. Chest auscultation revealed muffled heart sounds with an apical systolic murmur and diminished breath sounds. The remainder of the examination was unremarkable.

**Figure 1 -:**
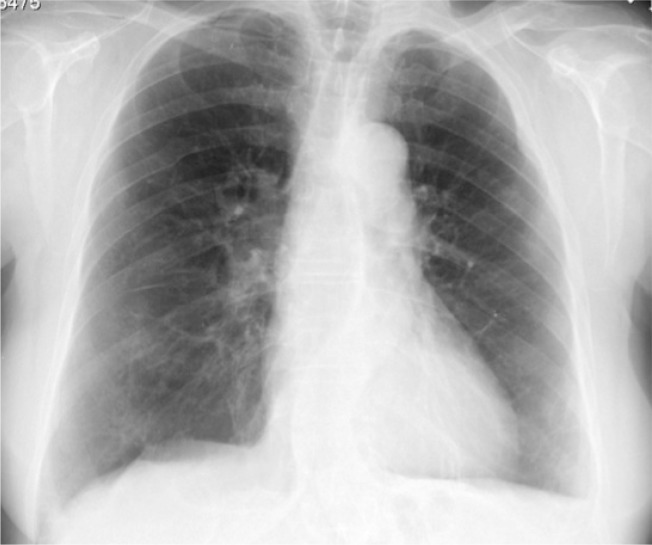
Chest X-ray at admission, showing a hypertransparent area in the upper mediastinum.

**Figure 2 -:**
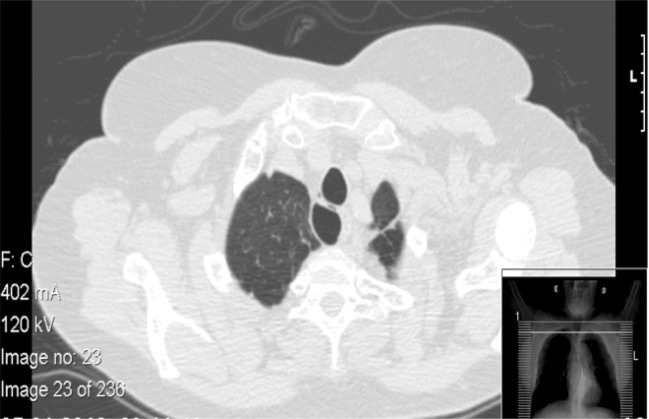
A CT scan of the chest at 10 days after admission, showing an air-filled multilocular mass (3.5 × 2.5 cm), with no evidence of infection, in the right wall of the trachea, near the apex of the lung.

**Figure 3 -:**
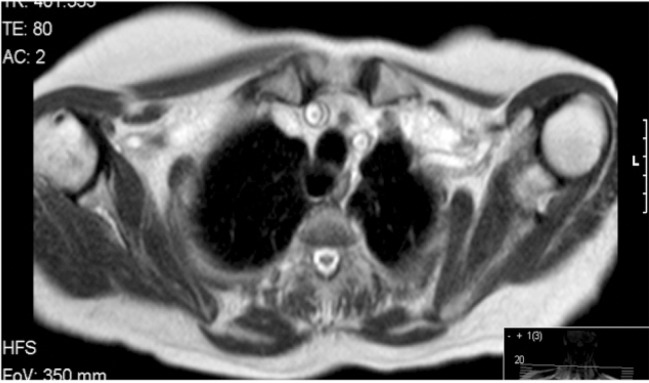
Cervical magnetic resonance imaging at 10 days after admission, showing the cystic image and the resolution of the infection.

Laboratory tests revealed elevated inflammatory markers (leukocytes at 18,300/µL, with neutrophilia, and C-reactive protein at 31.01 mg/dL) and mild hyponatremia (132 mmol/L). A chest X-ray (at admission) showed a hypertransparent area in the upper mediastinum (Figure 1), and a CT scan of the chest (performed 10 days after admission) showed an air-filled multilocular mass measuring 3.5 × 2.5 cm, with no evidence of infection, in the right wall of the trachea, near the apex of the lung (Figure 2). The CT scan also confirmed the pulmonary insufflation, panacinar emphysematous lesions, and bronchiectasis described in previous examinations. Other than hyperemia, fiberoptic bronchoscopy revealed no abnormalities, showing no unequivocal communication with the tracheal lumen. Nevertheless, a diagnosis of tracheal diverticulum was proposed. A cervical magnetic resonance imaging (MRI) scan confirmed the cystic image and the resolution of the infection (Figure 3).

After a 14-day course of meropenem and vancomycin, the patient showed clinical improvement. Her inflammatory markers returned to normal values, and she was discharged from the hospital. 

## Discussion

The scarcity of reports on tracheal diverticulum is due partly to its rarity and partly to the nonspecific nature of its symptoms, which tends to hinder the diagnosis.^(^ 2 ^-^ 5 ^,^ 8 ^,^ 9 ^,^ 11 ^,^ 14 ^)^ Tracheal diverticula are usually reported after the development of complications or in postmortem examinations. ^(^ 1 ^,^ 4 ^,6 9,^ 11 ^,^ 13 ^,^ 14 ^)^ The largest case series, reported by Goo et al.,^(^ 10 ^)^ consisted of 64 confirmed cases.

Here, we have reported a case of a tracheal diverticulum associated with respiratory tract infection, a right cervical mass, dysphagia and a two-month history of hoarseness. The patient had a history of recurrent respiratory infections and chronic pulmonary disease, specifically emphysema, which is known to be associated with tracheal diverticulum. However, to our knowledge, this is the first report of alpha-1 antitrypsin deficiency in a patient with tracheal diverticulum. It seems obvious that this was a case of acquired tracheal diverticulum, given the presence of emphysema and the history of thyroidectomy, which could have created weak points in the cervical anatomy, increasing the propensity to develop diverticula. In addition, although we did not perform a histological examination to confirm this hypothesis, the location of the tracheal diverticulum was that most often reported for the acquired form.^(^ 1 ^,^ 2 ^,^ 8 ^)^

The treatment approach to tracheal diverticulum varies according to the age of the patient, the clinical presentation, and the presence of comorbidities. Surgical resection is typically reserved for younger, highly symptomatic patients, whereas older patients, especially those with comorbidities, are treated conservatively.^(^ 12 ^)^ In the case presented here, the patient received conservative treatment. Her history of recurrent infection (and consequent antibiotic courses) was probably responsible for the lack of response to the initial treatment and the subsequent requirement to switch to broad-spectrum antibiotics. Although the definitive diagnosis was infected tracheal diverticulum, the CT and MRI, while still showing the air cyst, indicated resolution of the infection, because they were both performed after at least 10 days of treatment with antibiotics. The fiberoptic bronchoscopy missed the point of communication with the trachea and was not very helpful in the diagnosis, as has been previously reported. ^(^ 3 ^,^ 4 ^,^ 6 ^,^ 10 ^,^ 11 ^,^ 14 ^,^ 17 ^)^

In conclusion, tracheal diverticula are mostly asymptomatic and frequently go undiagnosed. When symptomatic, the approach to treatment should be tailored to the patient. In many cases, as in the one reported here, conservative treatment is indicated, although a surgical approach can be considered in patients who do not respond to clinical treatment.
